# ER Stress Mediates TiAl_6_V_4_ Particle-Induced Peri-Implant Osteolysis by Promoting RANKL Expression in Fibroblasts

**DOI:** 10.1371/journal.pone.0137774

**Published:** 2015-09-14

**Authors:** Zhenheng Wang, Naicheng Liu, Tongguo Shi, Gang Zhou, Zhenzhen Wang, Jingjing Gan, Ting Guo, Hongbo Qian, Nirong Bao, Jianning Zhao

**Affiliations:** Jinling Hospital, Department of Orthopaedics, School of Medicine and State Key Laboratory of Pharmaceutical Biotechnology, School of Life Sciences, Nanjing University, 210093, Nanjing, China; Faculté de médecine de Nantes, FRANCE

## Abstract

Wear particle-induced osteolysis is a major cause of aseptic loosening, which is one of the most common reasons for total hip arthroplasty (THA) failure. Previous studies have shown that the synovial fibroblasts present in the periprosthetic membrane are important targets of wear debris during osteolysis. However, the interaction mechanisms between the wear debris and fibroblasts remain largely unknown. In the present study, we investigated the effect of ER (endoplasmic reticulum) stress induced by TiAl_6_V_4_ particles (TiPs) in human synovial fibroblasts and calvarial resorption animal models. The expression of ER stress markers, including IRE1-α, GRP78/Bip and CHOP, were determined by western blot in fibroblasts that had been treated with TiPs for various times and concentration. To address whether ER stress was involved in the expression of RANKL, the effects of ER stress blockers (including 4-PBA and TUDCA) on the expression of RANKL in TiPs-treated fibroblasts were examined by real-time PCR, western blot and ELISA. Osteoclastogenesis was assessed by tartrate resistant acid phosphatase (TRAP) staining. Our study demonstrated that ER stress markers were markedly upregulated in TiPs-treated fibroblasts. Blocking ER stress significantly reduced the TiPs-induced expression of RANKL both in vitro and in vivo. Moreover, the inhibition of ER stress ameliorated wear particle-induced osteolysis in animal models. Taken together, these results suggested that the expression of RANKL induced by TiPs was mediated by ER stress in fibroblasts. Therefore, down regulating the ER stress of fibroblasts represents a potential therapeutic approach for wear particle-induced periprosthetic osteolysis.

## Introduction

Wear particles induced osteolysis and the subsequent aseptic loosening are the most common reason for arthroplasty failure and revision surgery [1]. Although fibroblasts constitute the majority of the cells in membrane interfaces, information on the response of fibroblasts to wear debris is less extensive than such information for other cell types such as macrophages and osteoclasts [2]. Recently, studies have reported that fibroblasts in the periprosthetic membrane are an important source of RANKL (Receptor activation of nuclear factor (NF)-kB). Generally, osteoclastogenesis is largely regulated by RANKL and OPG (osteoprotegrin) [3]. RANKL can promote osteoclast differentiation and activity while OPG is a decoy receptor that limits the biologic activity of RANKL, and the balance between RANKL and OPG is essential to regulate bone remodeling [4–7]. However, the mechanism underlying the expression of RANKL in fibroblasts that are affected by wear particles remains largely unknown [8].

Numerous studies have demonstrated that the endoplasmic reticulum plays a central role as the principal stress sensor through ER stress, also known as the unfolded protein response (UPR), in the regulation of cell energy metabolism, cell survival, inflammation and redox status [9–11]. ER stress is also involved in the differentiation of osteoclast precursor cells [12]. Moreover, Yip et al demonstrated that osteoclastogenesis was moderated by an ER stress inducer (thapsigargin) in a dose-dependent manner and that it depends on cross-talk with the RANKL signaling pathway [13]. Additionally, metal wear particles retrieved from patients undergoing aseptic loosening are found to be as small as 50 nm (range 6–834 nm) [14, 15]. And with the nano scale of particles, we demonstrated in a previous study that wear debris induced ER stress and mediated the expression of inflammatory cytokines in macrophages. Furthermore, ER stress markers were significantly upregulated in periosteum tissue from clinical specimens [16]. Given that the fibroblast is a major cell type in the periprosthetic membrane of patients, the questions that arise include whether ER stress is induced by wear particles in fibroblasts and the role of dose in the pathological process of particle-induced osteolysis. In the present study, we hypothesized that ER stress could be involved in particle-induced osteolysis through the upregulation of RANKL expression in fibroblasts. We tested this hypothesis in primary synovial fibroblast cells and a particle-induced calvarial osteolysis model.

## Material and Methods

### Reagents

Bovine serum albumin (BSA), Thapsigargin (Tg), Sodium tauroursodeoxycholate (TUDCA), 4-phenylbutyric acid (4-PBA), and protease inhibitor cocktail were purchased from Sigma-Aldrich (St. Louis, MO, USA). DMEM and were purchased from Hyclone (Logan, UT, USA).

### Cell culture

Synovial fibroblasts (SF) were harvested from surgical samples from osteoarthritis patients as previously described [17]. The experiment was approved by the Ethics Committee of Nanjing Jinling Hospital. Written informed consent was obtained from patients before fibroblasts were isolated. Cells were obtained by enzymatic digestion according to conventional protocols and cultured in DMEM medium supplemented with 10% FBS, penicillin (100 IU/ml) and streptomycin (100 μg/ml). Cells were maintained at 37°C in a humidified atmosphere of 5% CO_2_ and 95% air.

To explore the effect of TiPs on the expression of ER stress markers, fibroblasts were cultured with 1000 μg/ml TiPs for various times (0, 3, 6, 12, and 24 h) or were cultured with various concentration of TiPs (0, 50, 500 and 1000 μg/ml) for 24h. Cells that treated with thapsigargin (100 nmol/L) for 24 h was used as the positive control.

To address the effect of ER stress on the expression of RANKL and OPG, fibroblasts were exposed to 1000 μg/ml TiPs for various times (0, 3, 6, 12, and 24 h), various concentrations (0, 10, 100, and 1000 μg/ml) of TiPs for 12 h or various concentrations (0, 10, 50, and 100 nmol/L) of thapsigargin for 6 h. ER stress inhibitors 4-PBA (5 and 20 mmol/L) and TUDCA (100 μmol/L) were co-cultured with fibroblasts for 3h before being stimulated with TiPs (1000 μg/ml) for 12 h. The cells were collected to perform western blot and real-time PCR, and the supernatants were used for ELISA detection.

To obtain conditioned medium (CM), fibroblasts were seeded into 6-well plates at a density of 1 x 10^6^ cells/well. After 24h, the supernatant was removed and fresh medium containing 1000 μg/ml TiPs (with/without 5 mM 4-PBA and 100 μM TUDCA) was added. Cells cultured in fresh medium without TiPs were used as negative control. After 12 h, the conditioned medium from fibroblasts cultures were collected, centrifuged at 4000rpm for 15min, and then frozen at -20°C until use.

### Particle preparation

The TiAl_6_V_4_ particles with a mean particle diameter of 51.7 nm was described as previously [16]. The particles were suspended in phosphate buffered saline (PBS, pH 7.2–7.4) at a concentration of 50 mg/ml as a stock solution. The particles were autoclaved for 15 min at 121°C and 15 psi for sterilization. For in vitro experiments, the particles were ultrasonicated for 10 min before the cells were exposed to them. All of the particles were free of endotoxin, as determined by a quantitative Limulus Amebocyte Lysate (LAL) Assay (Charles River, Grand Island, UK) at a detection level of 0.25% EU/ml.

### In vivo calvarial resorption model

A wear particle-induced calvarial osteolysis model was generated as previously described [16]. All animals received humane care according to Chinese legal requirements. All the animal work and approach have been approved by the IACUC of the Nanjing Jinling Hospital. Briefly, 28 healthy 6-7-week-old C57BL/J6 mice with a mean weight of 21.1±1.1 g were assigned randomly into four groups: group I, sham-operated PBS mice; group II, TiPs-treated mice; group III, TiPs co-treated with 4-PBA mice; group IV, co-treated with TUDCA mice. The animals had free access to water and food and were kept in a 12 h on/12 h off specific pathogen-free animal room. The mice were anesthetized with an intraperitoneal injection of pentobarbital and the cranial periosteum was separated from the calvaria by sharp dissection. TiPs suspensions (40 μl of 30 mg/ml suspension) were embedded under the periosteum around the middle suture of the calvaria. Group I, which received 40 μl phosphate-buffered saline (PBS), was used as a sham control group. For group III and IV, 4-PBA (300 mg/kg) or TUDCA (200 mg/kg) was administered in sterile PBS intraperitoneally after surgery, and 4-PBA or TUDCA was injected daily for two weeks until the animals were sacrificed in a CO_2_ chamber. After the operation, no complications occurred and all wounds healed uneventfully. The calvarial caps were removed by dissecting the bone to free it from the underlying brain tissue.

### Micro-computed tomography (micro-CT) scanning

The mouse calvaria were scanned using a high-resolution micro-CT (SkyScan1176; SkyScan, Aartselaar, Belgium) at a resolution of 18 μm, and X-ray energy settings of 45 kV and 550 μA. A square region of interest (ROI) around the midline suture was selected for further qualitative and quantitative analysis after reconstruction. Bone volume/total volume (BV/TV) and percentage of total porosity within the ROI were obtained using the software provided with the micro-CT system, as reported previously [18].

### Histology and histochemistry

After micro-CT analysis, the toluidine blue staining assay was performed as described previously [19]. Briefly, bone slices were deposed in 0.25% trypsin for 15 min and were then left overnight in 0.25 M ammonium hydroxide before staining in 0.25% toluidine blue for 15 min. Slices were manicured and placed on slides with buffering glycerin after being washed vigorously and air dried. Osteoclasts were detected by immunohistochemical staining with tartrate resistant acid phosphatase (TRAP). The specimens were then observed and photographed with a light microscope.

### Bone histomorphometry

The area of bone resorption measurements were processed using Image Pro Plus software (Media Cybernetics, Silver Spring, MD, USA) as previously described [16, 19]. The bone resorption ratio (between the area of bone resorption and the area of its bounding box) was examined with photographed microscopic fields and the lacunar resorption ratio was calculated automatically.

### Immunofluorescence staining

The calvarial periosteum of the animals was permeabilized by 0.3% Triton X-100 and then blocked by adding a solution 5% BSA in PBS for 1 h. Slides were then incubated with primary antibody at 4°C overnight. A mouse monoclonal antibody against prolyl 4-hydroxylase (5B5; Abcam, MA, USA), and a goat polyclonal RANKL antibody (Santa Cruz Bio., CA, USA) were used. The slides were washed with PBS before incubation in FITC/TRITC labeled donkey anti-rabbit or FITC/TRITC labeled goat anti-mouse (KPL, Gaithersburg, MD, USA) antibodies. After incubation with DAPI for 5 min, the slides were captured with a Nikon confocal microscope (C2+, Nikon, Japan).

### ELISA for sRANKL detection

The sRANKL were quantified using enzyme-linked immunosorbent assay (ELISA) kits (BioVendor, Czech Republic). All procedures were performed according to the manufacturer's instructions.

### Western blotting

The cells prepared for western blotting were lysed in RIPA lysis buffer with a protein inhibitor cocktail for 30 min on ice. The lysates were centrifuged at 12,000 g for 10 min at 4°C, and the supernatants were collected. Protein concentration was determined using a BCA protein assay kit (Biocolor Bioscience and Technology Co., Shanghai, China).

Thirty micrograms of each protein were separated by 10% sodium dodecyl sulfate-polyacrylamide gel electrophoresis (SDS-PAGE) and then transferred to a polyvinylidene fluoride membrane (Pall Co., East Hills, NY, USA). Western blot was performed using the following primary antibodies: anti-inositol-requiring kinase 1 (IRE1-a), anti-glucose-regulated protein 78 (GRP/Bip), anti-C/EBP homologous protein (CHOP), and anti-glyceraldehyde 3-phosphate dehydrogenase (GAPDH) (Cell Signaling Technology, Beverly, MA, USA); anti-receptor activator of nuclear factor kappa-B ligand (RANKL) and anti- osteoprotegrin (OPG) (Santa Cruz, CA, USA); subsequently, the following secondary antibodies were applied: horseradish peroxidase-conjugated anti-rabbit IgG (Cell Signaling Technology) and horseradish peroxidase-conjugated anti-mouse IgG (Santa Cruz, CA, USA). After probing with a specific primary antibody and incubating with a horseradish peroxidase-conjugated secondary antibody, the protein bands were detected using a chemiluminescence detection system. The band density was analyzed using the ImageJ 1.41 software (National Institutes of Health, Bethesda, MD, USA).

### Real-time PCR

Total RNA from calvarial bone was prepared using TRIzol reagent (Invitrogen, 15596–018) according to the manufacturer’s instructions. Five micrograms total RNA was reverse transcribed to cDNA with the first-strand cDNA synthesis kit (Invitrogen, 18080–051). Real-time PCR was performed using 2 x SYBR Green qPCR Mix (Zoonbio Biotechnology Co., PC01) according to the manufacturer's protocol. Primers for β-actin were used as internal controls. The following primers were used: RANKL, sense: 5′-TGGGGCTCAATCTATATCTCGAAC-3’ and antisense: 5’-TGGATCACAGCACATCAGAGCAG-3′; OPG, sense: 5′-AGCTTGCACCACTCCAAATCC-3’ and antisense: 5’-GGGGACCACAATGAACAAGTTG-3′; GAPDH, sense: 5′- ACCACAGTCCATGCCATCAC-3’ and antisense: 5’- TCCACCACCCTGTTGCTGTA -3′.

### In vitro osteoclastogenesis assay

As described previously [20], primary bone marrow cells (BMMs) were isolated from the whole bone marrow of 15 healthy 4-week-old male C57BL/J6 mice. Briefly, BMMs were isolated from the femur bone marrow and cultured in complete α-MEM with 30 ng/ml M-CSF in a suspension culture dish at 37°C for 2–3 d. Nonadherent cells were removed, and adherent cells were released by 0.02% EDTA in PBS. Then the cells were seeded in 24-well plates and added with medium that containing half of conditioned medium from TiPs-treated or untreated fibroblasts and half fresh medium supplemented with 15% FBS. Positive control was added with 20 ng/ml recombinant RANKL (R&D Systems, Minneapolis, MN). Cell culture media were replaced every 2 days. All groups were treated with 30 ng M-CSF (PeproTech EC Ltd., London, UK). After 8–10 days, the cells were analyzed for TRAP activity (Jiancheng, Nanjing, China): TRAP-positive cells with three or more nuclei were considered as osteoclasts and been counted under a microscope.

### Flow cytometry analysis

Cells were pretreated with/without TUDCA (100 μM) for 3 hours before being stimulated with TiPs for another 24 hours. Subsequently, the cells were collected and stained with the Annexin V-FITC apoptosis detection kit (4A Biotech Co. Ltd, FXP018-100) according to the manufacturer’s instructions and analyzed by flow cytometry (FACSCalibur, BD. Bioscience).

### Caspase-3 activity analysis

Cells were incubated with/without TUDCA (100 μM) for 3 hours before being stimulated with TiPs for another 24 hours. Cell were lysed in lysis buffer (50 mM Tris-HCl (pH 7.4), 1 mM EDTA, 10 mM EGTA, 10 mM digitonin and 2 mM DTT). The cell lysates (150 μg proteins) were incubated with caspase-3 specific substrates at 37°C for 4 h. The fluorescence was measured with an enzyme-linked immunosorbent assay reader at OD_405_. All procedures were performed according to the manufacturer's instructions (Jiancheng, Nanjing).

### Statistical analysis

The results were expressed as the mean ± standard error of the mean (S.E.M). Kolmogorov-Smirnov test was used to assess normality. The differences among groups was analyzed by ANOVA, and comparison between two groups was analyzed using Dunnett’s test or Bonferroni test. A p-value less than 0.05 was considered as a significant difference.

## Results

### Activation of ER stress by TiPs in human synovial fibroblasts

**Fig 1:**
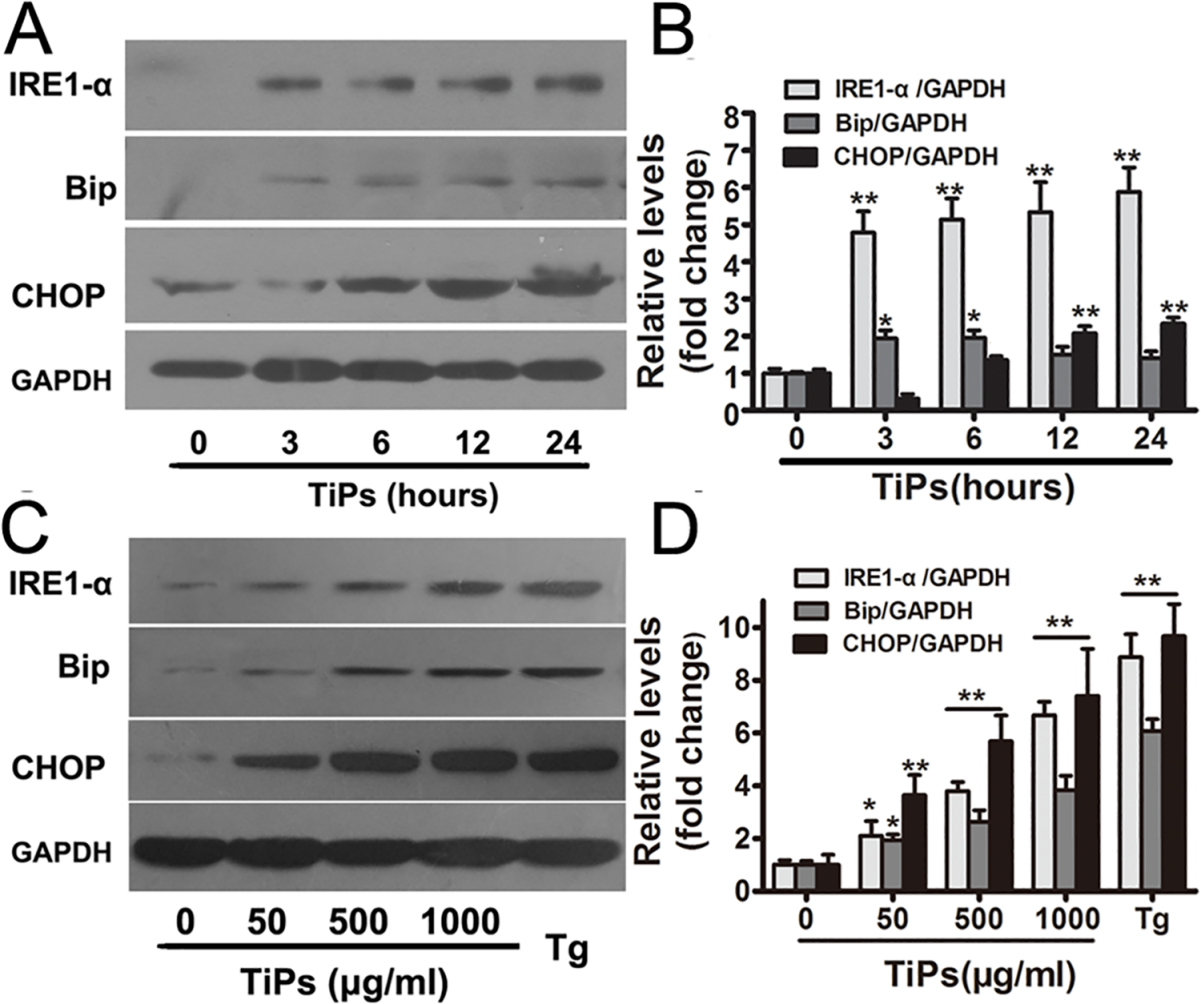
Activation of ER stress following exposure to TiPs in fibroblasts. (A and C) Western blots analysis of IRE1-α, Bip and CHOP in fibroblasts following treatment with TiPs for various time periods and concentrations. (B and D) The density of western blots bands shown in (A and C) were quantified using ImageJ software. Data are represented as the means ± S.E.M from three independent experiments. *P < 0.05, **P < 0.01.

Firstly, we detected the expression of ER stress markers in TiPs-stimulated fibroblasts. The results showed that TiPs markedly increased the protein levels of ER stress markers in both time- and dose-dependent manners (Fig 1A–1D). Compared with the control (time 0), the expression of IRE1-α induced by TiPs at 24 h was almost 6-fold higher (Fig 1A and 1B). The expression of CHOP induced by TiPs at 1000 μg/ml was almost 5.5-fold higher compared with the untreated cells (Fig 1C and 1D). These data suggested that TiPs could induce ER stress in primary fibroblasts.

### ER stress mediated the upregulation of RANKL and the RANKL/OPG ratio in TiPs-stimulated fibroblasts and promoted osteoclastogenesis in vitro

**Fig 2:**
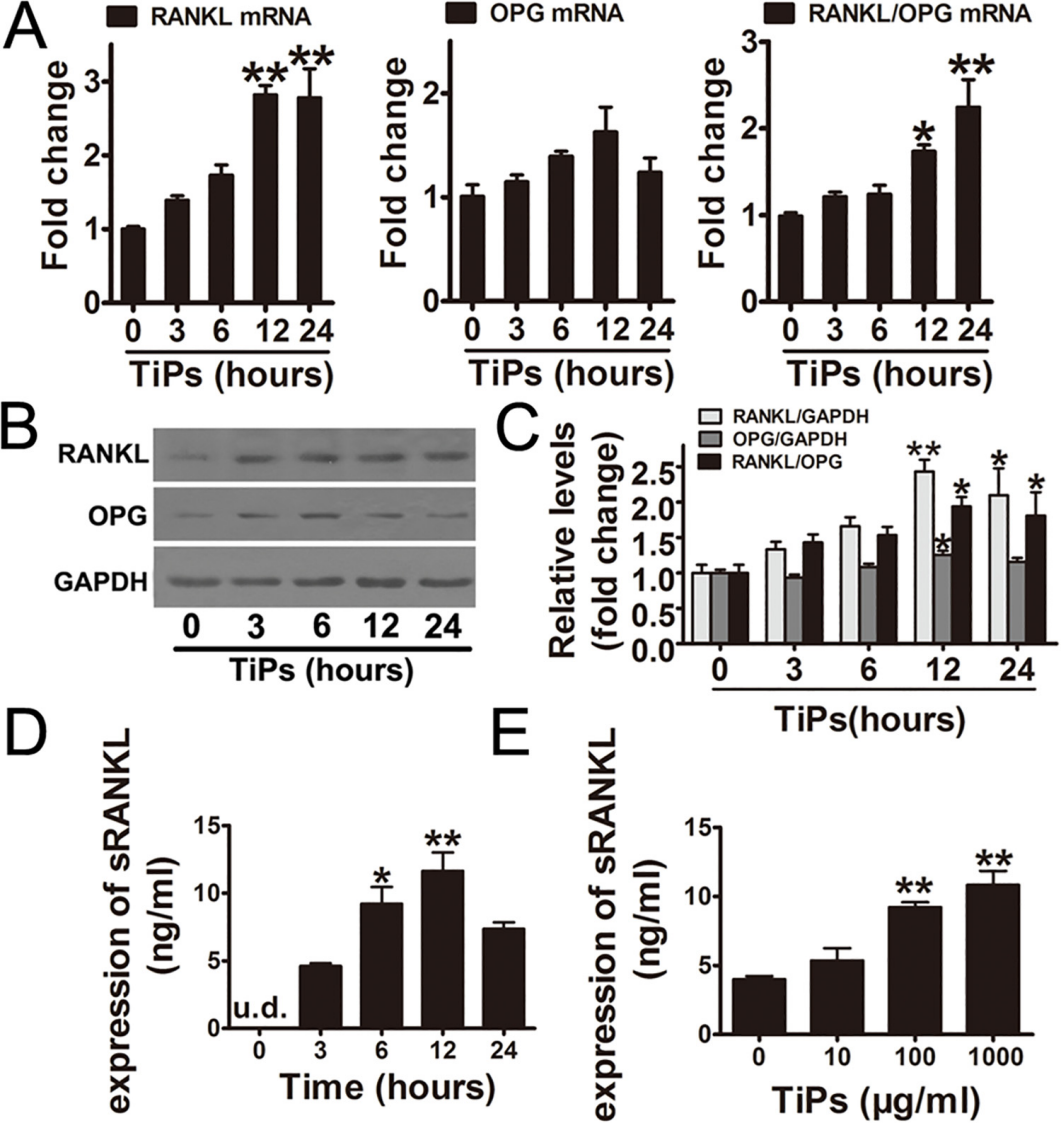
Induction of RANKL expression by TiPs. (A) The gene expression of RANKL and OPG in TiPs-treated fibroblasts were examined by real-time PCR. (B) Western blots analysis of RANKL and OPG in fibroblasts following treatment with TiPs for various time periods. (C) The density of western blots bands shown in (B) was quantified using the ImageJ software. (D and E) The expression of sRANKL in the supernatants from each group were quantified by ELISA. Data are represented as the means ± S.E.M from three independent experiments. (A and C) *P < 0.05, **P < 0.01 versus control (time 0). (D) *P < 0.05, **P < 0.01 versus time 3 (hours). (E) **P < 0.01 versus control (concentration 0).

In TiPs-treated fibroblast cells, we observed a time-dependent upregulation of RANKL mRNA expression (Fig 2A). The level of OPG mRNA did not significantly change although it had an increasing tendency within 12 h of exposure (Fig 2A). More importantly, the ratio of RANKL/OPG was increased in a time-dependent manner (Fig 2A). We also observed that the protein level of RANKL and the relative ratio of RANKL/OPG in the TiPs treatment were significantly increased compared with the control group (time 0) (Fig 2B and 2C). Furthermore, the sRANKL in supernatants was significantly upregulated by TiPs and reached a peak at 12 h (Fig 2D). Next, we found that TiPs significantly increased sRANKL expression in a dose-dependent manner (Fig 2E).

**Fig 3:**
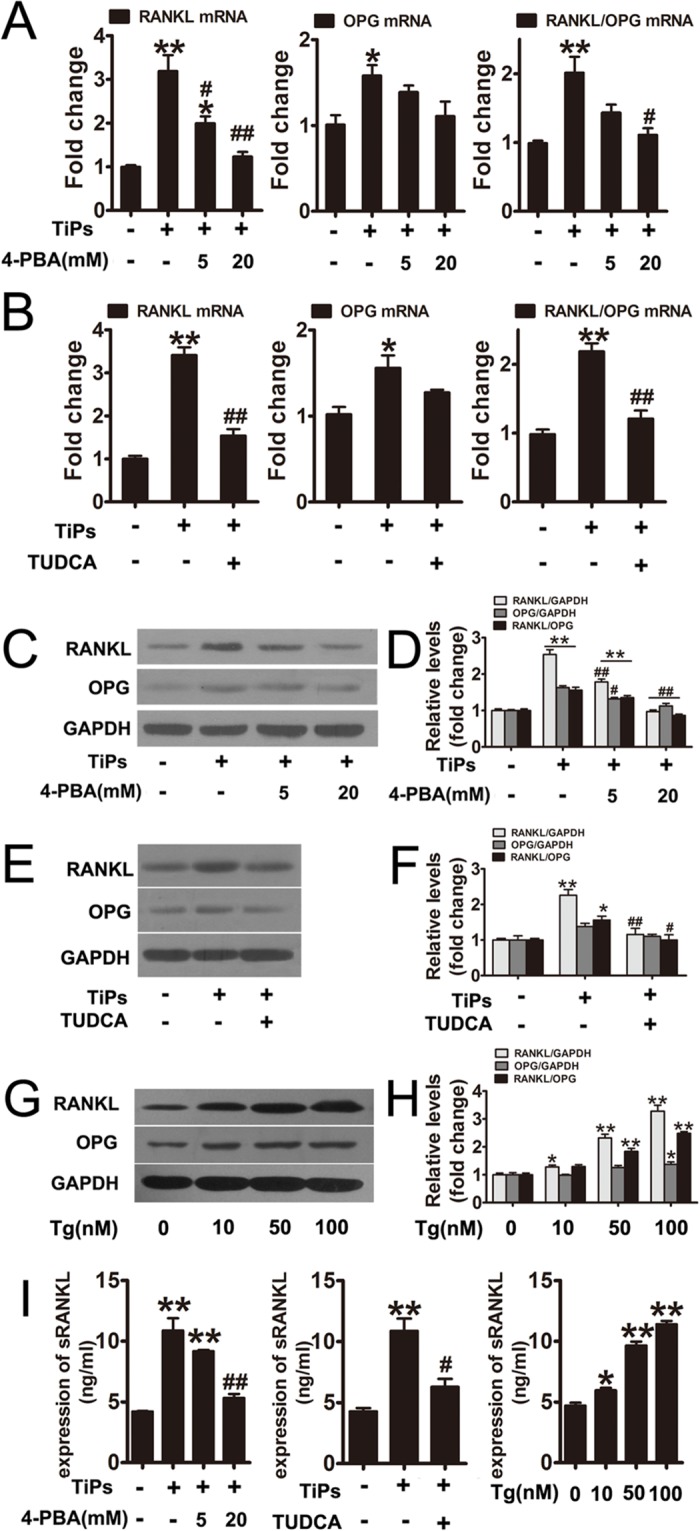
ER stress mediated the upregulation of RANKL and the RANKL/OPG ratio in TiPs-stimulated fibroblasts. (A and B) The gene expression of RANKL and OPG in fibroblasts from each group were examined by real-time PCR. (C, E and G) Western blots analysis of RANKL and OPG from each group. (D, F and H) The density of western blots bands shown in (C, E and G) was quantified using the ImageJ software. (I) The expression of sRANKL in the supernatants of fibroblasts from each group were quantified by ELISA. Data are represented as the means ± S.E.M from three independent experiments. *P < 0.05, **P < 0.01 versus control; #P < 0.05, ##P<0.01 versus TiPs group.

To investigate whether TiPs-induced RANKL and RANKL/OPG upregulation occurred as a result of ER stress, fibroblasts were pretreated with 4-PBA or TUDCA before stimulation with TiPs. The results showed that 4-PBA and TUDCA strongly inhibited RANKL mRNA and protein expression in fibroblasts while inhibiting ER stress (Fig 3A–3F). Compared with TiPs group, incubating cells with 4-PBA at 20 mmol/L was sufficient to cause a 60% and 40% loss of RANKL mRNA and protein expression, respectively (Fig 3A, 3C and 3D). The relative ratio of RANKL/OPG was significant decreased by both 4-PBA and TUDCA (Fig 3C–3F). In addition, the expression of sRANKL induced by TiPs was significantly inhibited by 4-PBA and TUDCA (Fig 3I). To further address the effect of ER stress on RANKL expression, fibroblasts were exposed to thapsigargin. The data demonstrated that thapsigargin induced both RANKL protein expression in cells and sRANKL expression in the supernatants (Fig 3G, 3H and 3I). These results suggested that ER stress was involved in TiPs-induced RANKL expression in fibroblasts.

**Fig 4:**
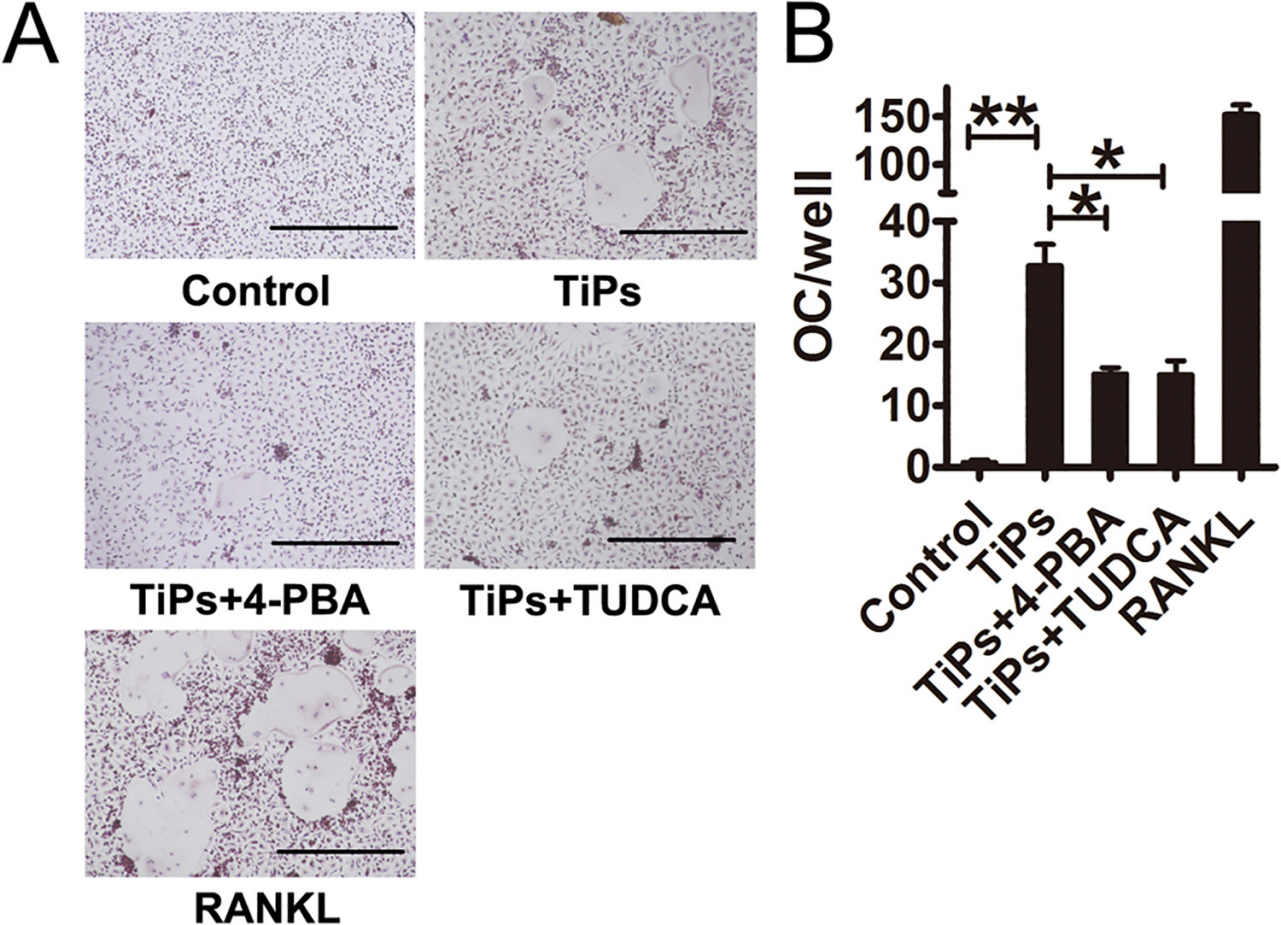
TiPs-induced ER stress in fibroblasts promoted osteoclastogenesis in vitro. (A) Bone marrow-derived macrophages (BMMs) were cocultured with conditioned medium (CM) from each group. Osteoclasts were detected by TRAP staining. Control, BMMs + untreated fibroblasts CM; TiPs, BMMs + TiPs-treated fibroblasts CM; TiPs + 4-PBA, BMMs + TiPs and 4-PBA co-treated fibroblasts CM; TiPs + TUDCA, BMMs + TiPs and TUDCA co-treated fibroblasts CM; RANKL, BMMs + RANKL. Scale bar, 500 μm. (B) The number of osteoclasts in (A) were analyzed. OC, osteoclast.

To determine the functional significance of ER stress on RANKL expression, an in vitro osteoclastogenesis assay was performed. Data revealed that osteoclast formation was markedly impaired in the 4-PBA and TUDCA co-treated groups when compared with TiPs group (Fig 4A). The mean numbers of multinucleated TRAP+ cells were summarized in Fig 4B.

As ER stress maybe involved in apoptosis, we examined cell apoptosis in fibroblasts. The apoptosis of the cells was not significantly changed by TiPs or TUDCA (S1A and S1B Fig). These results were further confirmed by the examination of caspase-3 activity (S1C Fig) in fibroblasts.

### Inhibition of ER stress ameliorated TiPs-induced osteolysis and osteoclastogenesis in a murine calvaria resorption model

**Fig 5:**
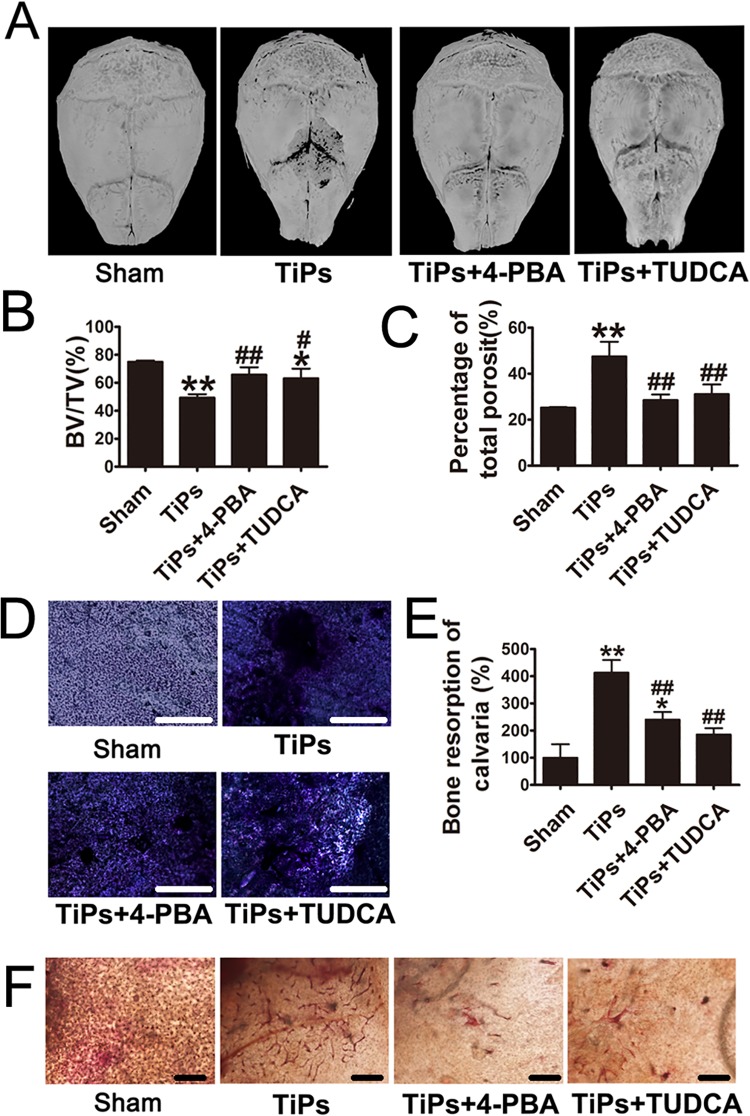
ER stress inhibitors ameliorated TiPs-induced mouse calvarial osteolysis and osteoclastogenesis. (A) Representative micro-CT with three-dimensional reconstructed images from each group. (B and C) Bone volume/total volume (BV/TV) and percentage of total porosity of each sample were measured. (D) Toluidine blue staining of calvaria derived from each group. Scale bar, 1mm. (E) Bone resorption of calvaria (%) in (D). (F) Histochemical staining with TRAP of calvaria derived from each group. Scale bar, 25 μm. Data are presented as the mean± S.E.M. n = 7 mice per group. *P < 0.05, **P < 0.01 versus sham; #P<0.05, ##P<0.01 versus TiPs group.

As shown in Fig 5A, the degree of bone resorption in TiPs-induced calvaria was much higher than that in the sham group. However, treatment with 4-PBA or TUDCA significantly suppressed the osteolysis induced by TiPs (Fig 5A). A quantitative analysis of bone parameters further confirmed that both 4-PBA and TUDCA significantly rescued the BV/TV and decreased the percentage of total porosity compared to the TiPs-treated group (Fig 5A–5C).

Histological and histochemical assessment further confirmed the attenuation of particle-induced bone resorption by inhibiting ER stress. TiPs implantation resulted in a large number of toluidine blue-positive lacunas in the calvarial caps (Fig 5D), which indicated that marked bone resorption had occurred. Consistent with the micro-CT analysis, histochemical analysis demonstrated that 4-PBA and TUDCA significantly reduced osteolysis induced by wear particles (Fig 5D). Co-treatment with TUDCA decreased the bone resorption ratio by 60% in TiPs-treated mice (Fig 5E). Next, we investigated whether the effect of bone resorption caused by ER stress was related to an increase in osteoclasts. The results of histochemical staining with TRAP, which was used as a cytochemical marker for osteoclasts, were in good agreement with the results of toluidine blue-staining (Fig 5F). Co-treatment with ER stress inhibitors decreased the osteoclastogenesis in TiPs-treated mice.

### ER stress mediated the upregulation of RANKL of fibroblasts in vivo

**Fig 6:**
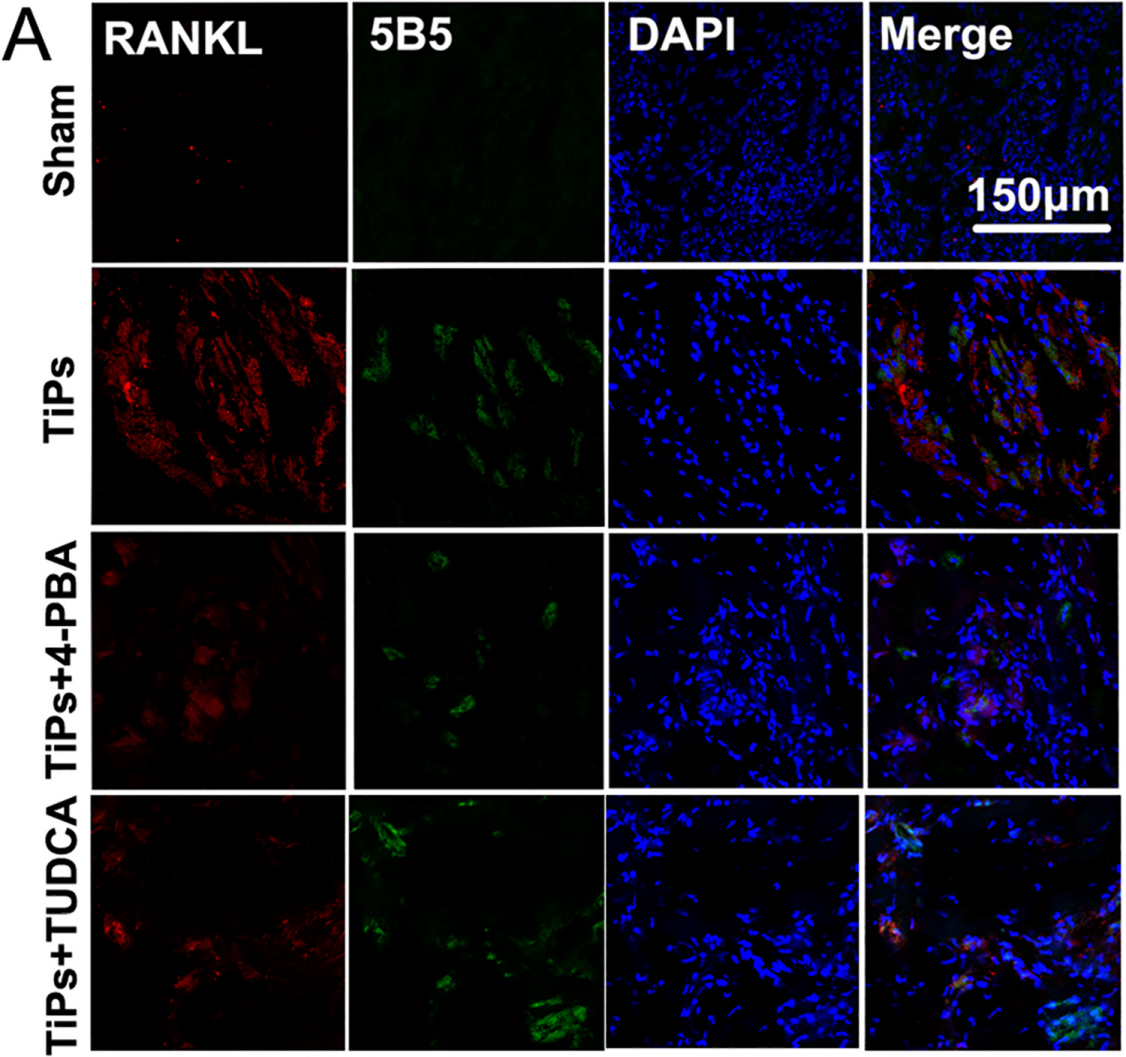
ER stress mediated the upregulation of RANKL of fibroblasts in vivo. (A) Immunofluorescence was performed to determine the expression of RANKL in fibroblasts. Murine calvarial periosteum is shown for animals from each group. Red, RANKL; green, fibroblasts (5B5); blue, DAPI nuclear staining. Scale bar, 150 μm.

To further investigate the relationship between ER stress and RANKL expression in fibroblasts in animal models, we analyzed the expression of RANKL in fibroblasts in the calvarial periosteum. As shown by the immunofluorescence depicted in Fig 6A, massive RANKL expression was observed in TiPs-treated mice. Both the expression of RANKL and the number of fibroblasts were markedly increased in the TiPs-treated group compared with the sham group. More importantly, the induction of RANKL by particles was inhibited by co-treatment with 4-PBA or TUDCA. Collectively, these data implied that ER stress mediated the upregulation of RANKL secretion in fibroblasts in this particle-stimulated murine calvaria resorption model.

## Discussion

One of the pathologic features of aseptic loosening is the formation of a fibrous synovia-like membrane in the gap between the prosthesis and bone. This membrane interface consists of multiple cell types, including fibroblasts, macrophages, T lymphocytes, and foreign body giant cells [21–23]. These cells proliferate and secrete various cytokines, including TNF-α, RNAKL, IL-6, IL-1β, IL-8 and matrix metalloproteinases (MMPs), which lead directly to bone resorption and indirectly to the activation of osteoclasts [24–27]. Fibroblasts, which compose 70% of the cells in the membrane, can markedly promote the expression of proinflammatory cytokines, collagenase and stromelysin, which contribute to the bone resorption process [2, 28]. Fibroblasts isolated from membrane interface of patients with aseptic loosening could induce osteoclast differentiation from monocytes [23]. Fibroblasts have also been identified as a major cell type that is responsible for expression of RANKL in the periprosthetic membrane [5]. RANKL plays a key role in the recruitment, differentiation and activity of the osteoclasts implicated in peri-implant osteolysis. The blockade of RANKL signaling in murine models effectively ameliorated titanium particle-induced osteolysis [29–31]. Several studies have demonstrated that wear debris increased RANKL expression in fibroblasts [32, 33]. Titanium particles significantly increased the RANKL expression in synovial fibroblasts [28]. The induction of RANKL by PE particles was observed both in primary mouse fibroblasts and in a mouse model [34]. However, the mechanism underlying the expression of RANKL induced by wear particles remains largely unknown [8]. In the present study, we demonstrated that Ti-alloy particles markedly increased the expression of RANKL and the ratio of RANKL/OPG in fibroblasts. Interestingly, the inhibition of ER stress markedly decreased the expression of RANKL and the ratio of RANKL/OPG in fibroblasts. The effect of ER stress on RANKL expression in fibroblasts was further confirmed by the ER stress inducer.

Our current experiments showed that the presence of TiPs dramatically induced ER stress in a time- and dose-dependent manner in fibroblasts. Previously, studies have showed that various nanomaterials induced ER in vitro and in vivo [35–39]. Nanogold particles induced ER stress in K562 cells [40]. Zinc oxide nanoparticles caused increased ER stress markers, which may responsible for the upregulation of pro-apoptotic proteins (including caspase-3, caspase-9 and Bax) and hepatotoxicity in mice [39]. Nanoparticles can be uptaken by cells and accumulate in ER over time [41], which may disturb the physiological functions of ER and result in the ER stress. In addition, nanomaterials can induced the production of ROS (reactive oxygen species), which may lead to ER stress indirectly [36].

ER stress has been associated with a wide range of diseases, including neurodegeneration, inflammatory disease, cardiac disease, cancer, diabetes and others [11]. Previously, we demonstrated that ER stress contributed to the induction of proinflammatory cytokines in macrophages [16]. ER stress was also revealed to be involved in osteoclastogenesis [12, 13]. An inducer of ER stress (thapsigargin) has been shown to induce osteoclast formation from RAW264.7 macrophages [13]. The inhibition of ER stress leads to downregulation of MCP (monocyte chemotactic protein)-1-mediated osteoclast precursor differentiation [12]. In the present study, the inhibition of ER stress significantly decreased the formation of osteoclasts that induced by TiPs in vitro and in vivo. The data further confirmed the important role of ER stress in osteoclastogenesis. Further, co-treatment with ER stress inhibitors decreased TiPs-induced RANKL expression and decreased the relative ratio of RANKL/OPG. In animal models, TiPs-induced RANKL expression in fibroblasts was inhibited by the ER stress inhibitors. As RANKL is the key final effector of osteoclastogenesis, these results indicated that the promoting role of ER stress on osteoclastogenesis was mainly by upregulation of RANKL expression.

In the present study, the Ti-alloy particles we used were nanoparticles because that metal wear particles isolated from the periprosthetic tissue of patients were mainly nano-sized [14, 15]. In fact, the sizes of the wear debris in vivo were found to vary among the materials used in prosthetics [15]. For example, UHMWPE (ultra-high-molecular-weight polyethylene) debris in vivo were found to be larger in size (micro-size) than metal debris [15]. However, various sizes particles were reported to result in osteolysis and aseptic loosening in patients [42–44]. The phenomenon may due to that particles of different materials with various size are able to stimulate the secretion of inflammatory mediators which act locally at the site of cell infection and damage [15]. Both nano- and micro-sized particles can induced the upregulation of RANKL, TNF-α, IL-6, IL-1β in vitro and in vivo [15, 16, 45, 46]. On one hand, the inflammatory mediators increased the formation and activity of osteoclast and lead to the upregulation of peri-implant bone resorption [16]. On the other hand, the inflammatory cytokines may inhibition the osteoblast proliferation and differentiation and lead to the downregulation of bone formation [21]. The complex and sustained local cellular response disturbs the balance of bone metabolism and results in peri-implant osteolysis. Thus, particles with various size cause aseptic loosening in clinical [15]. Although the pathologic process was similar clinically, the precise mechanism may be different. In our previous study, inflammatory cytokines induced by nano-sized metal particles were mediated by ER stress in macrophages, however, micro-sized polymethylmethacrylate (PMMA) particles did not induced ER stress in vitro (data are not shown) [16].

In addition to inhibiting RANKL expression in fibroblasts in vitro and in vivo, we demonstrated that an inhibitor of ER stress potently inhibited osteolysis induced by wear particles in vivo, as assessed by both micro-CT and histological analysis. These results indicated that blocking of ER stress in fibroblasts could represent a potential therapy for wear particle-induced osteolysis.

Collectively, our results demonstrated that ER stress mediated the expression of RANKL in fibroblasts, and downregulating ER stress in fibroblasts may represent a potential therapy for wear particle-induced osteolysis.

## Supporting Information

S1 Fig Fibroblast apoptosis was not affect by TiPs and the ER stress inhibitor. (A) Flow cytometry analysis of Annexin V and propidium iodide staining of fibroblast cells from each group. (B) Quantification analysis of apoptotic cells in (A) (both upper- and lower-right quadrants in representative dot plots as shown). Data are represented as means ± S.D. from three independent experiments. (C) Caspase-3 activity was measured in fibroblasts from each group. Data are represented as means ± S.E.M. from three independent experiments. n.s., no significance.
(TIF)

S1 File The ARRIVE guidelines for reporting animal studies. (PDF)
