# Supplementary figures and images for: ER Stress Mediates TiAl6V4 Particle-Induced Peri-Implant Osteolysis by Promoting RANKL Expression in Fibroblasts
Source: PLoS One. 2015 Sep 14;10(9):e0137774. doi: 10.1371/journal.pone.0137774 (PMC4569331; doi:10.1371/journal.pone.0137774)

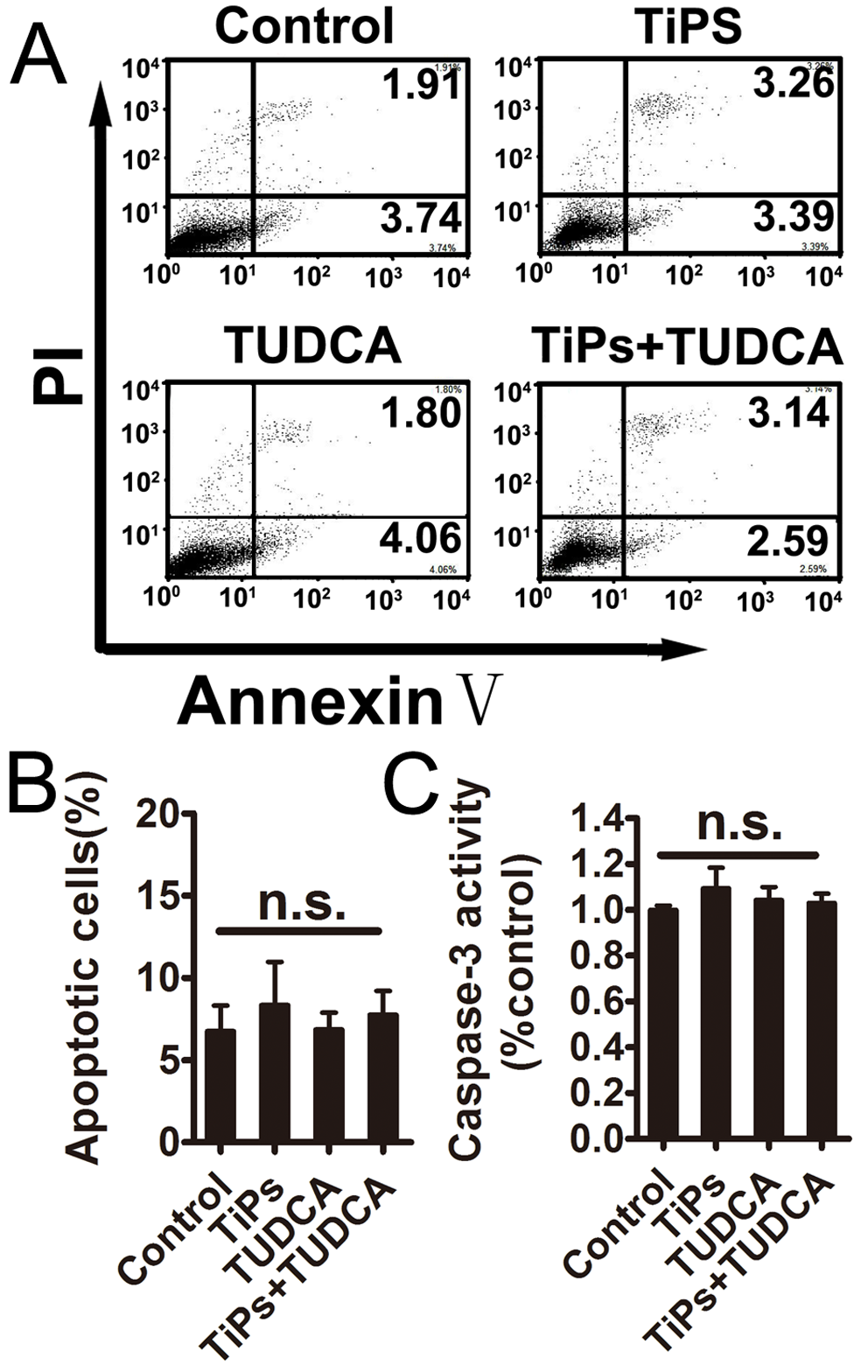

Supplement: S1 Fig — (A) Flow cytometry analysis of Annexin V and propidium iodide staining of fibroblast cells from each group. (B) Quantification analysis of apoptotic cells in (A) (both upper- and lower-right quadrants in representative dot plots as shown). Data are represented as means ± S.D. from three independent experiments. (C) Caspase-3 activity was measured in fibroblasts from each group. Data are represented as means ± S.E.M. from three independent experiments. n.s., no significance. (TIF) [file pone.0137774.s001.tif]
